# Which Stereotest do You Use? A Survey Research Study in the British Isles, the United States and Canada

**DOI:** 10.22599/bioj.120

**Published:** 2019-02-13

**Authors:** Kathleen Vancleef, Jenny C. A. Read

**Affiliations:** 1 Newcastle University, GB

**Keywords:** stereopsis, binocular vision, assessment, Randot stereotest, Frisby, TNO

## Abstract

A wide range of stereotests are available to measure stereopsis. Because each test has its own advantages and disadvantages, opinions differ on which is the preferred test to use in clinical practice. We conducted surveys comparing the use of stereotests in the British Isles and in the United States and Canada.

Two online surveys were developed following consultation with eye care professionals, one for each geographical area. Both surveys included two questions on the frequency of use of different stereotests, two questions on best practice stereotests, and two questions on the usefulness of stereotests. Researchers made distinctions between appointments with children below or above 6 years old for respondents from the British Isles and below or above 5 years old for respondents from the Unites Stated and Canada. The surveys were distributed through professional organisations.

We found Frisby to be the most used stereotest on the British Isles for both age groups. In the US and Canada, Titmus and Randot stereotest are more frequently used. Respondents consider these tests as the best practice stereotests. Eye care professionals agree stereotests are useful in the diagnosis and treatment decision making and even more so in obtaining an accurate measure of stereoacuity, especially with older children.

## Introduction

Stereo vision or stereopsis is the ability to perceive the relative depth of objects based on binocular disparity, which refers to the small difference in angles between images of objects in left and right eyes. Poor stereopsis is often linked to strabismus and amblyopia (Levi, Knill and Bavelier 2015; Read 2015). With misaligned eyes, as in strabismus, the object’s images on the left and right retina can be too far apart to fall within the range of fusion matches that can be achieved by the brain. Also, good visual acuity in each eye is essential to achieve stereopsis, which is problematic in anisometropic amblyopia (Levi, Knill and Bavelier 2015). Because stereo vision depends upon good vision in both eyes, excellent oculomotor control and the development of binocular brain mechanisms, a measurement of stereoacuity is often regarded as the gold standard for binocular visual function (Elliott and Shafiq 2013).

Stereopsis is regularly measured to inform diagnosis and decision making in treatment of amblyopia and strabismus (Fricke and Siderov, 1997a; Elliott and Shafiq, 2013; Ciner et al., 2014). A range of stereotests are commercially available, each with their own advantages and disadvantages (for comparisons see for example Cooper, Feldman and Medlin, 1979; Simons, 1981; Ohlsson et al., 2001; Garnham and Sloper, 2006; Leske, Birch and Holmes, 2006). Because the results from different tests are not necessarily comparable (Cooper, Feldman and Medlin 1979; Simons, 1981; Garnham and Sloper 2006; Leske, Birch and Holmes 2006; Hahn et al., 2010) the choice of stereotest is important. The choice can be guided by a preference to measure local or global stereopsis. Global stereopsis refers to a process of cross-correlation of the left and right image while local stereopsis refers to extracting depth information from monocularly-visible contours by facilitating vergence or qualitative depth judgements (Vancleef et al., 2017). Global stereopsis is measured with random dot stereograms like TNO (Lameris, Ede, Netherlands) or Preschool Randot (Stereo Optical, Chicago, IL, US) (Watanabe et al., 2014). This is believed to be particularly sensitive to conditions like strabismus and amblyopia (Sloper and Collins 1999; The Royal College of Ophthalmologists, 2012). Local stereopsis is measured with contour stereograms like Randot Circles and Animals (Stereo Optical) and Titmus Fly (Stereo Optical). The age of the child is another important factor in the choice of stereotest. The purpose of testing, screening or obtaining a threshold measure to monitor treatment, also guides the choice of stereotest (Fricke and Siderov 1997b). The choice of test is often guided by practical considerations such as costs and the availability of tests within the eye department.

We are not aware of any reports on the use of different stereotests and whether there is consensus on the best practice stereotest between eye care professionals. We conducted surveys to evaluate the use of stereotests, best practice recommendations and usefulness of stereotests in the British Isles and in the Unites States and Canada.

## Methods

A survey was designed to evaluate the use of stereotests by eye care professionals in the British Isles and in the US and Canada.

Initial survey questions and statements for clinical needs were identified during discussions with an orthoptist, an ophthalmologist, optometrists, and stereo vision researchers. Early versions of the questionnaire were circulated within this expert group to assure face validity. The questionnaire was subsequently validated with 25 practicing orthoptists from different eye departments in the North of England. Questions were amended based on the comments received. We made minor changes to the wording of questions, and adjusted the answer options of the multiple choice questions to make it more relevant for the respondents.

We designed two versions of the final survey, one for respondents within the British Isles and one for respondents in the US and Canada. The two versions show small differences (see Table 1) reflecting differences in how eye care and specifically strabismus and amblyopia is managed on the different continents. For instance, the British Isles questionnaire was targeted at orthoptists, while the US and Canadian questionnaire was opened up for optometrists and ophthalmologists too. The US and Canadian survey included filter questions to ensure only questions relevant for a particular respondent were displayed. For instance, only respondents who indicated that they worked in the US were given the option to select which state they were practicing in.

**Table 1:** Survey questions.

Survey questions	Response options

Which of the following best describes your current occupation? (US and Canadian survey only)	Optometrist Ophthalmologist Optician Orthoptist Other (please specify): Open-Ended Response
In your current job as an eye specialist, do you see children during your appointments? (filter question, US and Canadian survey only)	Yes (proceed to next question) No (exit survey)
Where do you currently work? (filter question, US and Canadian survey only) Please state your Country of work (British Isles survey only)	The US (proceed to question on the US states and territories) Canada (proceed to question on Canadian provinces and territories) Other (please specify): Open-Ended Response Open-Ended Response for British Isles survey
In what state or US territory do you currently work? (US and Canadian survey only) OR In what province or Canadian territory do you currently work? (US and Canadian survey only)	The US states and territories OR Canadian provinces and territories
Out of 10 appointments with children between 3 and 6 years old, how many times do you normally use the following stereotests? (US and Canadian survey: age range 3–5) - Frisby - Randot (British Isles survey only) - Randot Stereotest (incl circles and animals) (US and Canadian survey only) - Randot Preschool Stereotest (random dot stereograms) (US and Canadian survey only) - Titmus - TNO - Random dot E - Lang I or II - Other (please specify)	Never Between 1 and 3 times out of 10 Between 4 and 6 times out of 10 Between 7 and 9 times out of 10 Always Open-Ended Response for ‘Other’
Out of 10 appointments with children between 6 and 12 years old, how many times do you normally use the following stereotests? - Frisby - Randot (British Isles survey only) - Randot Stereotest (incl circles and animals) (US and Canadian survey only) - Randot Preschool Stereotest (random dot stereograms) (US and Canadian survey only) - Titmus - TNO - Random dot E - Lang I or II - Other (please specify)	Never Between 1 and 3 times out of 10 Between 4 and 6 times out of 10 Between 7 and 9 times out of 10 Always Open-Ended Response for ‘Other’
Which stereotest do you consider best practice for measuring stereoacuity in children between 3 and 6 years old? (US and Canadian survey: age range 3–5)	Open-Ended Response
How useful do you think this stereotest is for children between 3 and 6 years old - in obtaining an accurate measure of stereoacuity - in diagnosis - in decision making on treatment (US and Canadian survey: age range 3–5)	Not at all useful Slightly useful Somewhat useful Very useful Extremely useful
Which stereotest do you consider best practice for measuring stereoacuity in children between 6 and 12 years old? How useful do you think this stereotest is for children between 6 and 12 years old - in obtaining an accurate measure of stereoacuity - in diagnosis - in decision making on treatment	Open-Ended Response Not at all useful Slightly useful Somewhat useful Very useful Extremely useful
Free comments section	Open-Ended Response

The survey questions can be grouped into biographical questions on professional role and location (1 question in the British Isles survey, 4 questions in the US and Canadian survey), frequency of use of different stereotests (2 questions, might be limited by availability of stereotests), best practice stereotests (2 questions, not limited by availability of stereotests), and usefulness of stereotests (2 questions). For all questions on stereotesting a distinction was made between children between 3 and 6 years old (5 years for US and Canadian survey) and older children. The difference in age range between the British Isles and US and Canadian survey reflects cultural difference in clinical practice highlighted by US optometrists involved in the design of the survey. The questions are presented in Table 1. All questions were optional. The surveys were made available online through SurveyMonkey.

The questionnaires were distributed through professional organisations (BIOS British and Irish Orthoptic Society, AAPOS American Association for Pediatric Ophthalmology and Strabismus, Canadian Association of Optometrists, American Association of Certified Orthoptists, The Canadian Orthoptic Society), personal contacts, and social media (Twitter, Facebook, and LinkedIn) between August 2015 and December 2015 for the British Isles survey and between June 2016 and November 2016 for the US and Canadian survey.

Data were collected anonymously and participants were informed about the purpose of the research, voluntary participation, right to withdraw at any time, and the procedures in place to ensure anonymity. All respondents consented before taking part in the survey. The study protocol was compliant with the Declaration of Helsinki and was approved by the Ethics Committee of the Newcastle University Faculty of Medical Sciences (reference 00888).

In preparation for data-analyses, common themes in the responses to open-ended questions were identified and responses were coded. All p-values for Chi^2^ test statistic were generated through Monte Carlo simulations (n = 2000) to account for small numbers of expected frequencies in certain categories. To account for multiple comparisons, alpha was adjusted via Bonferroni correction and set at 0.005 (standard alpha of 0.005 divided by 11 Chi^2^ tests) for the analyses of British Isles and US/Canada data independently and set at 0.004 (standard alpha of 0.05 divided by 14 Chi^2^ tests) for the comparison between both geographical areas.

## Results

### British Isles survey

**Respondents’ profile.** We had 298 respondents on the British Isles survey. After excluding respondents who reported to practice outside the British Isles, 289 entries remained. 249 of these were from the United Kingdom: 122 from England, 16 from Scotland, 10 from Wales, 6 from Northern Ireland, and 95 did not specify their country. We had 9 respondents from Ireland, 1 from the Channel Islands, and 1 from Isle of Man. The remaining 29 respondents did not report their location. We assumed they were from the British Isles as that is where the survey has been advertised. This is a good reflection of the distribution of eye clinics over the different countries in the British Isles compared to previous reports (Chi^2^ = 0.24, p = 0.97, Gillespie-Gallery, Conway and Subramanian, 2012).

**Response rate.** At the time of the survey, BIOS had 1272 members who were practising orthoptists or assistants managed by an orthoptist (personal communication with BIOS). Only a proportion of these practitioners managed amblyopia and strabismus and were therefore eligible to take part. No report on the number of orthoptists providing these services is available, so no exact estimate of the response rate can be made, but based on the BIOS member information, we achieved an estimated response rate of at least 22.7%.

**Figure 1:**
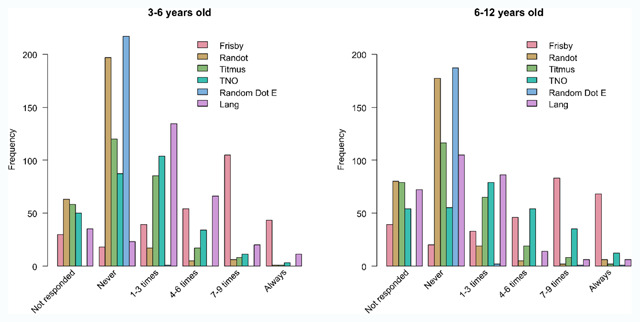
Bar charts of the use of each stereotest in appointments with 3- to 6-year-old children (left plot) and 6- to 12-year-old children (right plot); British Isle respondents only. Respondents indicate how often they used each stereotest out of 10 appointments with children of each age group.

**Frequency of use of stereotests.** Frisby (Frisby Stereotests, Fulwood, United Kingdom) is used most often by orthoptists on the British Isles: 41% of the respondents use the test 7 to 9 times out of 10 in appointments with children between 3 and 6 years old (Figure 1). TNO and Lang (Lang-Stereotest. Kusnacht, Switzerland) are used in 1 to 3 out of 10 appointments with this age group (44% and 53% respectively). Titmus, Randot (Stereo Optical), and Random dot E (Stereo Optical) are less frequently used. They are never used by 52% of respondents for the Titmus, 88% for the Randot, and 100% for the Random dot E. Other tests that were mentioned were Frisby-Davis distance stereotest (4 respondents, Frisby Stereotests), Distance Randot (1 respondent, Stereo Optical), Lang 2 Pen test (4 respondents), and Preschool Randot (1 respondent).

The results for children between 6 and 12 years old follow that same pattern with the exception of the Lang stereotest that is less frequently used in older children compared to younger children (Figure 1, Frisby: Chi^2^ = 9.29, p = 0.05; Randot: Chi^2^ = 6.10 p = 0.19; Titmus: Chi^2^ = 2.18, p = 0.71; Random dot E: Chi^2^ = 2.79, p = 0.33; Lang: Chi^2^ = 103.55, p < 0.01) and the TNO that is used a bit more often in the older age group (TNO: Chi^2^ = 33.06, p < 0.01).

**Best practice stereotests.** In both age groups, the Frisby stereotest is considered the best practice stereotest by most respondents (see Table 2), followed by TNO. TNO is more often recommended for older children than for younger children, while Lang is more often recommended for younger than older. A Chi square test indicated a significant difference between both age groups (Chi^2^ = 62.5, p < 0.01).

**Table 2:** Percentage of British Isles respondents that named the listed stereotest as the best practice stereotest.

Stereotest	For 3–6 years old (n = 269)	For 6–12 years old (n = 270)

Frisby	68.4	53.0
TNO	13.0	40.4
Lang I or II	9.3	1.9
Titmus	6.7	2.2
Randot	2.2	2.2
Frisby-Davis 2	0.4	0.4
Random Dot E	0.0	0.0

**Figure 2:**
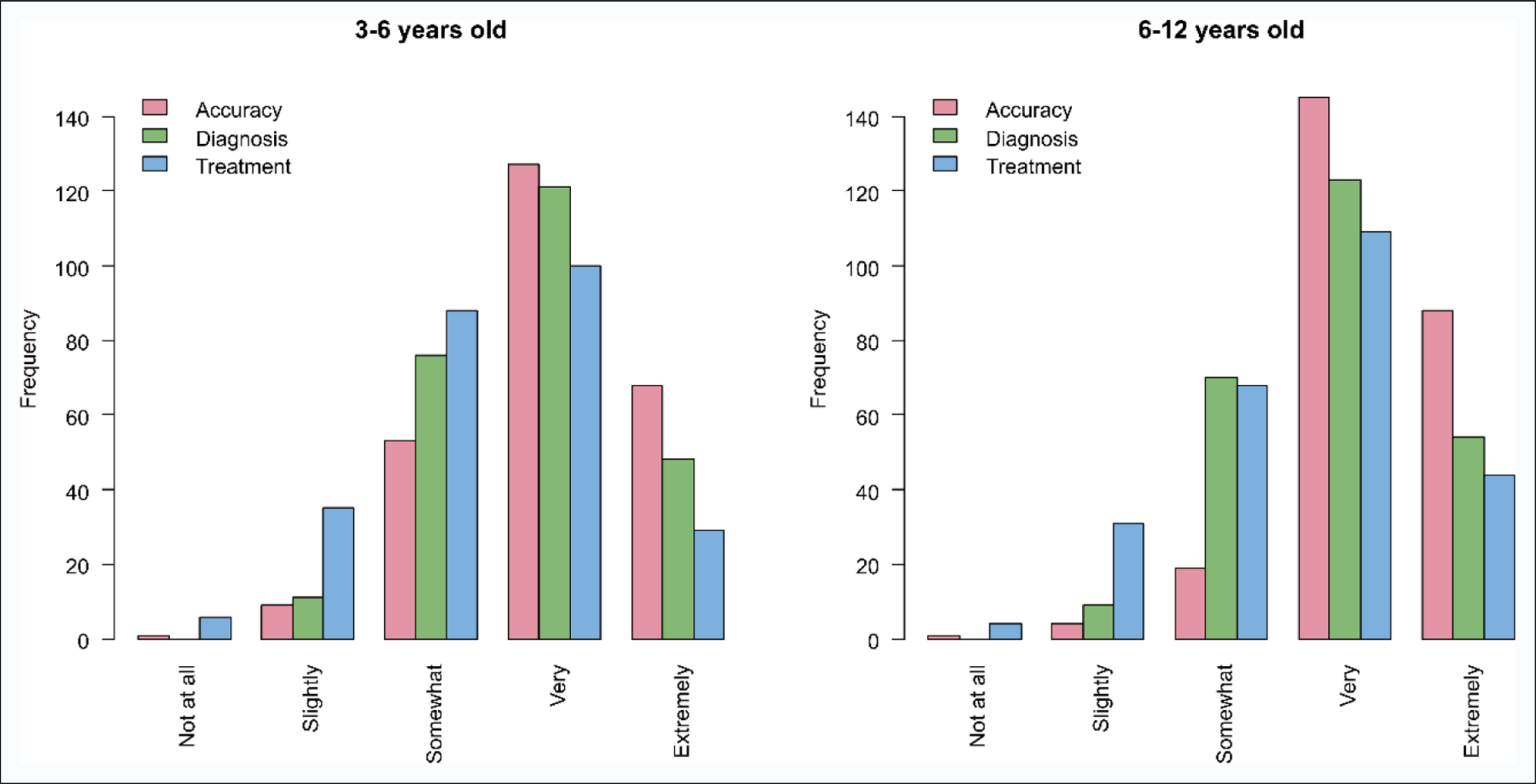
Usefulness of the best practice stereotest in obtaining an accurate measure of stereopsis (in red), in diagnosis (in green), and in decision making for treatment (in blue) for children between 3 and 6 years old (left) and between 6 and 12 years old (right).

**Usefulness of stereotests.** Most respondents rate their chosen best practice stereotest as being very or extremely useful in obtaining an accurate stereothreshold, in diagnosis and in decision making on treatment (Figure 2), although usefulness is considered higher in obtaining an accurate stereothreshold than in diagnosis or treatment decision. Opinions are similar in both age groups when it comes to usefulness in diagnosis and treatment decisions (diagnosis: Chi^2^ = 0.82, p = 0.86; treatment: Chi^2^ = 6.67, p = 0.18), but differ for usefulness in getting an accurate measure (Chi^2^ = 21.73, p < 0.01) with more reports of ‘extremely useful’ and ‘very useful’ for older children compared to younger children.

### US and Canadian survey

**Respondents’ profile.** 193 respondents entered the US and Canadian survey. Respondents with a location outside the US or Canada were excluded, as well as respondents who reported not seeing any children as an eye care professional and respondents who only completed the biographical questions. 167 entries remained. Our respondents included 62 ophthalmologists, 50 optometrists, 52 orthoptists, and 3 respondents who reported ‘other’ as their current occupation. 140 were from the United States and 27 from Canada. The US respondents were practicing in 38 different states with 1 to 14 respondents from each state. 19 of the 27 Canadian respondents came from Ontario, the other respondents had their practice in Alberta, British Columbia, Manitoba, Newfoundland and Labrador, or Nova Scotia.

**Response rate.** Because the survey was distributed by several professional bodies, we had no control over the number of members it was sent out to and how many of the people who received the invitation were eligible to participate. In addition, respondents might have received multiple invitations. Therefore the response rate cannot be calculated.

**Figure 3:**
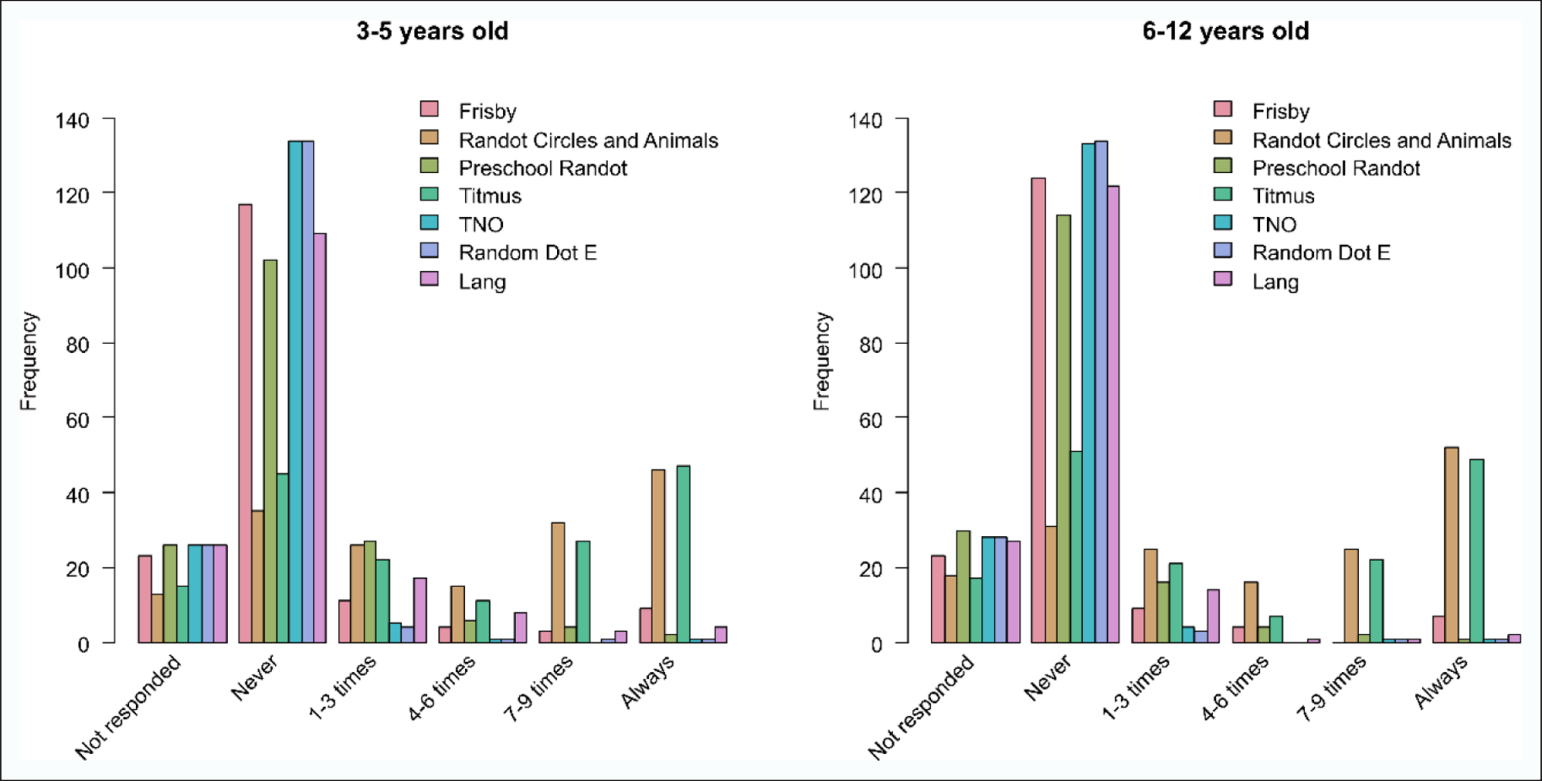
Bar charts of the use of each stereotest in appointment with 3- to 6-year-old children (left plot) and 6- to 12-year-old children (right plot). US and Canadian respondents only indicate how often they used each stereotest out of 10 appointments with children of each age group.

**Frequency of use of stereotests.** Titmus Fly and Randot Circles and Animals are the most popular stereotests in the US, with Titmus Fly being used all the time by 31% of the respondents and Randot Circles and Animals by 30% for children between 3 and 5 years old (Figure 3). The other stereotests are never used by most respondents: 81% never use Frisby, 72% never use Preschool Randot, 95% never use TNO, 95% never use Random Dot E, and 77% never use the Lang stereotest. Other tests that were mentioned were Worth’s Four Dot test (1 respondent), distance vectographic projector slide (1 respondent), Butterfly stereotest (2 respondents, Stereo Optical), ability to touch the flashlight with one finger (1 respondent), Keystone Basic Binocular Test (1 respondent, Keystone View, Reno, NV, US), and Preschool Assessment of Stereopsis with a Smile stereotest (1 respondent, Vision Assessment Corporation, Elk Grove Village, IL, US).

The results for children between 6 and 12 years old follow the same pattern, with Randot Circles and Animals and Titmus being the most frequently used stereotests (Figure 3, Frisby: Chi^2^ = 3.65, p = 0.49; Randot Circles and Animals: Chi^2^ = 1.44, p = 0.84; Preschool Randot: Chi^2^ = 4.82, p = 0.32; Titmus: Chi^2^ = 1.83, p = 0.86; TNO: Chi^2^ = 2.10, p = 1; Random dot E: Chi^2^ = 1.13, p = 1; Lang: Chi^2^ = 8.13, p = 0.07). Other tests that were used for this age group were Worth’s Four Dot test (1 respondent), distance vectographic projector slide (1 respondent), Butterfly stereotest (1 respondent), and the Lang pen test (1 respondent).

**Best practice stereotests.** In both age groups, the Randot stereotest is considered the best practice stereotest by most respondents (see Table 3), followed by Titmus. Many respondents did not specify which Randot test they considered best practice, therefore Randot Circles and Animals and Preschool Randot are merged into one category. A significant difference between age groups was observed (Chi^2^ = 18.23, p = 0.01), with Randot being recommended more often for older children than for younger children.

**Table 3:** Percentage of US and Canadian respondents that named the listed stereotest as the best practice stereotest.

Stereotest	For 3–5 years old (n = 143)	For 6–12 years old (n = 145)

Randot	49.7	60.7
Titmus	32.9	28.3
Frisby	7.7	2.1
Lang I or II	5.6	0.7
TNO	0	4.1
Random Dot E	2.1	2.1
Random Dot E	0.7	0.7
Butterfly stereotest	0.7	0.7
Stereo Reindeer test (Stereo Optical)	0.7	0.7

**Figure 4:**
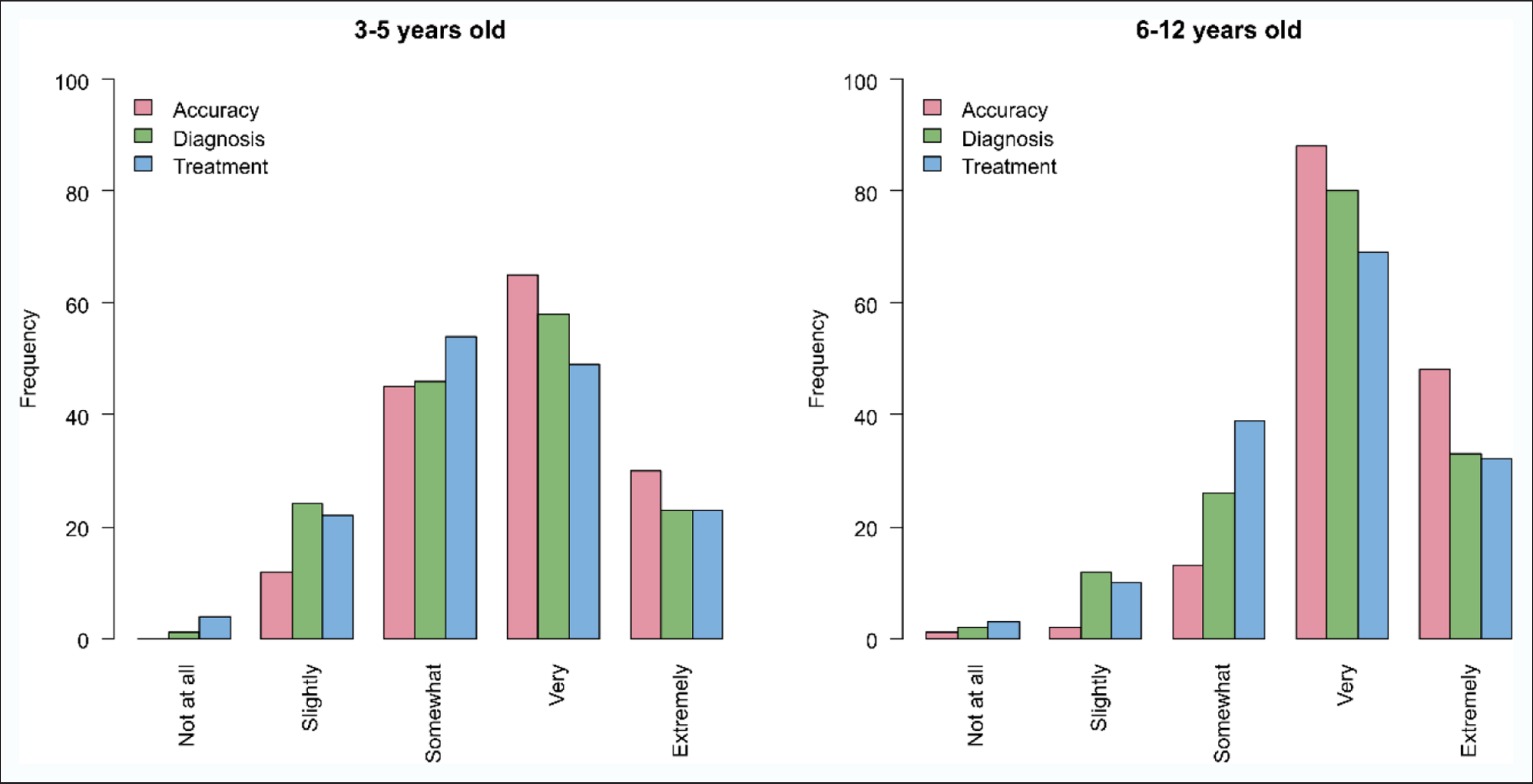
Usefulness of the best practice stereotest in obtaining an accurate measure of stereopsis (in red), in diagnosis (in green), and in decision making for treatment (in blue) for children between 3 and 5 years old (left) and between 6 and 12 years old (right).

**Usefulness of stereotests.** Most respondents rate their chosen best practice stereotest as being ‘somewhat’ or ‘very’ useful in obtaining an accurate stereothreshold, in diagnosis and in decision making on treatment for the younger children, and ‘very’ or ‘extremely’ useful for older children (accuracy: Chi^2^ = 33.41, p < 0.001; diagnosis: Chi^2^ = 15.182, p = 0.01; treatment: Chi^2^ = 11.92, p = 0.03). Stereotests are considered more useful in obtaining an accurate stereothreshold than in diagnosis or treatment decision (Figure 4).

### Comparison of British Isles versus US and Canadian survey

**Frequency of use of stereotests.** Because previous analyses (see above) indicate minimal difference between the frequency of use of stereotests in the different age groups, the data for both age groups are collapsed. On the one hand, we observe a significant higher use of the Frisby, Lang and TNO stereotest on the British Isles compared to the US and Canada (Frisby: Chi^2^ = 481.45, p < 0.001; Lang: Chi^2^ = 215.66, p < 0.001; TNO: Chi^2^ = 304.18, p < 0.001). On the other hand, the Randot stereotests (combined Preshool and Randot Circles) are significantly more popular in the US and Canada than on the British Isles (Chi^2^ = 164.46, p < 0.001). The use of the Titmus stereotest differs significantly between geographical areas. In the US most respondents report to either always (n = 96) or never (n = 96) use the test, while in the British Isles most respondents use the test never (n = 236) or only 1 to 3 times out of 10 (n = 150) (Chi^2^ = 209.81, p < 0.001). The Random dot E is rarely used in both geographical areas (Chi^2^ = 6.88, p = 0.11).

**Best practice stereotests.** We observed a significant difference between the British Isles versus the US and Canada in what is considered the best practice stereotest for younger and older children (younger children: Chi^2^ = 257.61, p < 0.001; older children: Chi^2^ = 325.66, p < 0.001). In line with the findings on the frequency of use of stereotests, respondents in the British Isles more frequently report the Frisby stereotest as the best practice test, while the Randot is more often reported as the preferred test in the US and Canada (for both age groups).

**Usefulness of stereotests.** Respondents in both geographical areas agree that their best practice stereotest is ‘very useful’ in obtaining an accurate measure of stereoacuity in young children (Chi^2^ = 10.11, p = 0.03) and in older children (Chi^2^ = 0.58, p = 0.97). Respondents from the British Isles found their best practice stereotest significantly more useful in diagnosis than their colleagues from the US and Canada for younger children (Chi^2^ = 18.9, p < 0.001) but not for older children (Chi^2^ = 11.57, p = 0.02). With respect to treatment, respondents agree that their best practice stereotest is ‘somewhat’ to ‘very useful’ for younger (Chi^2^ = 2.41, p = 0.64) and older children (Chi^2^ = 3.95, p = 0.40).

## Discussion

Our results reveal several interesting discrepancies in clinical practice regarding stereotests. For example, in the United Kingdom, Frisby is the most widely used stereotest for children between 3 and 6 years old and children between 6 and 12 years old. For the younger age group, this is followed by the Lang, while for the older children, the TNO is the second most popular stereotest. This does not agree with what eye care professionals consider the best practice stereotest. For both age groups, Frisby is mentioned by most respondents, followed by TNO, while Lang is only mentioned by few respondents. A potential explanation is that eye care professionals would prefer to use either Frisby or TNO with younger children, but more often resort to Lang because of limited cooperation in very young children.

In the United States and Canada, the choice of stereotests is very different. Eye care professionals most often choose the Titmus or the Randot Circles and Animals stereotest in their appointments with both younger and older children. Frisby, TNO, or Lang are rarely used. The reported use agrees with what these professionals consider to be the best practice stereotests.

Respondents to both the British Isles and US and Canada survey indicated their best practice stereotest is ‘somewhat’ or ‘very’ useful in diagnosis and treatment decisions. The US and Canadian respondents thought usefulness increases in the older age group, while British Isles respondents did not report a difference between age groups. When it comes to obtaining an accurate measure of stereopsis, opinions in the British Isles and the US and Canada agree that their best practice stereotest is ‘very’ useful.

The differences in preferred stereotest reported by our participants are consistent with advice from professional bodies in different geographical areas. In the US, the American Academy of Ophthalmology recommends the Randot stereotest in their Preferred Practice Pattern Guidelines for amblyopia and pediatric eye evaluations (American Academy of Ophthalmology Pediatric Ophthalmology/Strabismus Panel 2017a and 2017b), although the National Expert Panel to the National Center for Children’s Vision and Eye Health recommends the use of the Preschool Assessment of Stereopsis with a Smile (PASS, Vision Assessment Corporation) stereotest if stereopsis is measured in vision screening (Cotter et al., 2015). On the British Isles, the Royal College of Ophthalmologists (UK, 2012) recommends measuring near stereopsis with TNO, Frisby, Randot, Lang or a synoptophore in their guidelines for the management of strabismus and amblyopia. They do not make a distinction between the quality of different tests. Our survey indicates most health care professionals follow their professional body in the stereotest they consider best practice. The same pattern is also reflected in research work published in journals from the different regions. For example, the British and Irish Orthoptics Journal has published one paper referring to TNO and one to Frisby, but none referring to Randot. In contrast, the Journal of the American Association for Pediatric Ophthalmology and Strabismus includes more papers on Randot (43) than on TNO (16) or Frisby (26) (searches performed on 14/01/2019).

Our study cannot reveal the reasons for these geographical differences. We can distinguish three broad possibilities: (i) There are genuine reasons why one test is better in North America and another in the British Isles (e.g. different populations or practice arrangements). (ii) One stereotest is in fact the best everywhere, and some region(s) are using an inferior test. (iii) TNO, Frisby and Randot are all equally good and it does not matter which is used. We are not aware of any evidence in favour of (i). We suspect that the reasons for the differences in preferred stereotest between the two sides of the Atlantic have more to do with tradition than any clinically relevant difference. The Frisby stereotest was originally designed in the UK and consequently distributed and promoted more in the British Isles than in the US and Canada. Similarly the TNO stereotest was designed in a neighbouring country, the Netherlands. The Randot stereotests on the other hand were first brought to the market by a US based company, Stereo Optical.

Clearly, an ideal test would avoid non-stereo artefacts, such as the motion artefacts visible monocularly in both the Frisby and Lang stereotests if the patient moves their head. It also seems desirable to avoid the interocular colour differences introduced with anaglyph glasses, which may be responsible for the poorer stereo thresholds obtained with the TNO stereotest (Vancleef et al., 2017). However, the question of which stereotest is “best” is complicated by the fact that, as indicated in the Introduction, different tests measure different aspects of stereopsis. One key distinction is between local and global stereopsis, which are believed to be mediated by different neuronal populations and which are differentially affected by conditions such as amblyopia (Frisby et al., 1975; Giaschi, Lo and Narasimhan, 2013; Read 2015). If one is testing whether a subject has completely normal stereoscopic vision, we would recommend a cyclopean stimulus such as found in the Preschool Randot or Random Dot E stereotest, since such stimuli are regarded as “unfakeable” and “a very pure test of stereopsis” (Frisby et al., 1975). If instead one is looking for any residual stereopsis in a patient with a binocular vision disorder, one should use a test with clear monocular contours, e.g. Randot circles or animals.

The lack of agreement on the best practice stereotests across geographical areas has several potentially negative implications. Stereothresholds of different stereotests are not comparable, and as just noted, different tests measure different aspects of stereopsis. This might lead clinicians using different stereotests to reach different conclusions regarding a child’s stereopsis, which can have an effect on treatment options and reports of incidence of stereoblindness. It may also make it harder to build links between research and clinical practice: for example, evidence collected in one geographical area may not be directly applicable to areas where different stereotests are used. In research, if authors and reviewers are from different geographical areas, consensus on appropriate methods might be difficult to reach. In clinical practice, difficulties may arise for patients and professionals moving countries, something that is more frequent in our current global society. It would be helpful to reduce the transatlantic differences in practice noted by our study.

### Study limitations

A response rate of 22.7% or higher can be seen as a potential limitation of our study. However, this is a good response rate in comparison with previously published survey research in this population. A survey on low vision services in the British Isles reached a response rate between 9.6 and 17% (Gillespie-Gallery, Conway and Subramanian 2012). A survey on AC/A ratio reached 16% of the targeted population (Murray and Newsham, 2014), and a survey on the use of atropine penalization reached 151 orthoptists (estimated response rate of 10–15%) (Piano, O’Connor and Newsham 2014). In addition, the location of practice reported in our British Isles survey is a good reflection of the distribution of eye clinics over the different countries in the British Isles. A previous study reports 120 eye clinics in England, 13 in Scotland, 8 in Wales, and 2 in Northern Ireland (Gillespie-Gallery, Conway and Subramanian 2012). As reported in the results, our sample includes 122 respondents from England, 16 from Scotland, 10 from Wales, and 6 from Northern Ireland, indicating we have collected a geographically representative sample. The response rate for the US and Canadian survey could not be estimated. However it is likely to be lower given the lower number of respondents and the higher population. It is likely that Canadian eye care professionals were underrepresented in our sample, especially because the majority of the Canadian respondents practiced in Ontario.

Another possible limitation with especially the US and Canadian survey is the categorisation of stereotests. Over the years, several Randot stereotests have been developed with variations between different manufacturers. Each of these differ slightly in which subtests they include. The original Randot Stereotest presents the child with shapes in random dot stereograms, circles in diamonds, and animals in random dot stereograms (Stereo Optical). The most common Randot Stereotest (Stereo Optical) includes contour stereograms with circles in rows and animals and random dot stereograms with shapes (Stereo Optical). In other editions, the animals are replaced by Lea symbols (Vision Assessment Corporation: Random Dot 2 LEA SYMBOLS Stereoacuity Test). The next generation of Randot tests only includes random dot stereograms (Vision Assessment Corporation: Random Dot 3S Stereoacuity Test, Random Dot 3 LEA SYMBOLS Stereoacuity Test). The Preschool Randot (Stereo Optical, Vision Assessment Corporation) is a book with three pages, each presenting random dot stereograms at different disparities. To increase confusion, the Titmus stereotest also includes circles in diamonds and animals besides a Fly. Furthermore, the fly test is also known as the Wirth Fly or Fly stereotest (Stereo Optical: Original Stereo Fly Stereotest). Recent editions also include Lea symbols instead of animals (Vision Assessment Corporation: Stereopsis Fly with LEA SYMBOLS). Also, The Stereopsis Butterfly test (Stereo Optical and Vision Assessment Corporation) includes circles in diamonds besides animals/Lea symbols and a contour butterfly. It is likely that there was considerable difference between respondents in their awareness of these variations. This means that the labels “Randot” or “Titmus” stereotest could have been interpreted in different ways by different respondents. We see elements of this in the answers to the open-ended question on the best practice stereotest: we received many times the responses ‘Randot’, Randot Stereoacuity’, or ‘Randot stereo’ without any specification, ‘Animals’ without a reference to Randot or Titmus, ‘Randot Fly’ or ‘any of the Randot tests’, besides also clear answers like ‘Randot butterfly’, ‘Randot animals/circles’, or ‘Randot Preschool’. Since the Randot tests are most popular in the US this has most likely affected the responses to the US and Canada survey more that the British Isles survey.

Future research in this area can further look into the use of multiple stereotests in one patient. For instance, do practitioners look at convergent results from multiple stereotests, do they have multiple attempts with a range of stereotests in challenging patients, etc. Furthermore, the reason for using stereotests can be explored further as the choice of stereotest can vary depending on the symptoms of the patients.

### Summary

In conclusion, the current study describes the use of stereotests for children above and below 6 years old in the British Isles and in the United States and Canada. We observed a higher use of Frisby and TNO in the British Isles, while the Titmus and Randot are the most frequently used stereotest in the US and Canada. Eye care professionals agree stereotests are useful in the diagnosis and treatment decision making and even more so in obtaining an accurate measure of stereoacuity, especially with older children.
